# Primary Epstein–Barr virus infection diffusing F^18^-fluorodeoxyglucose-positron emission tomography/computed tomography response monitoring of Hodgkin’s disease: a case report

**DOI:** 10.1186/1752-1947-8-212

**Published:** 2014-06-20

**Authors:** Hans Balink, Mels Hoogendoorn

**Affiliations:** 1 Department of Nuclear Medicine, Medical Center Leeuwarden, P.O. Box 850, 8901 BR Leeuwarden, The Netherlands; 2 Department of Haematology, Medical Center Leeuwarden, P.O. Box 850, 8901 BR Leeuwarden, The Netherlands

**Keywords:** Autologous stem-cell transplantation (ASCT), Epstein–Barr virus (EBV), F18-FDG positron emission tomography/computed tomography (PET/CT), Hodgkin’s disease (HD)

## Abstract

**Introduction:**

Hodgkin’s disease is highly curable by radiotherapy and/or chemotherapy, but refractory disease or early relapses are rarely cured by conventional salvage therapy.

**Case presentation:**

We report a case of a 20-year-old Caucasian man, with a biopsy-proven intrapulmonary relapse of Hodgkin’s disease, for whom salvage chemotherapy was administered. During salvage chemotherapy intense increased F^18^-fluorodeoxyglucose uptake was noticed in multiple lymph nodes and diffuse increased splenic uptake, suggesting chemotherapy-refractory disease. However, additional information obtained from the patient revealed he recently had met his first girlfriend. An asymptomatic primary Epstein–Barr virus infection was considered proven.

**Conclusions:**

Interim F^18^-fluorodeoxyglucose-positron emission tomography/computed tomography is a strong prognostic factor for advanced Hodgkin’s and may better identify those patients needing intensified chemotherapy. Related to the nonspecificity of F^18^-fluorodeoxyglucose, clinical awareness of the potential interference of intercurrent asymptomatic viral infections with treatment and remission status monitoring continues to be important in the interpretation of equivocal medical imaging results.

## Introduction

Hodgkin’s disease (HD) is highly curable by radiotherapy and/or chemotherapy, but refractory disease or early relapses are rarely cured by conventional salvage therapy [1].

Additional high-dose chemotherapy (HDCT), such as carmustine, etoposide, cytarabine and melphalan (BEAM) in combination with autologous stem-cell transplantation (ASCT), is used to salvage and cure these patients. Since only chemosensitive patients with relapsed HD benefit from ablative therapy and ASCT, adequate response to salvage chemotherapy, assessed by serial positron emission tomography/computed tomography (PET/CT) is pivotal to progress to HDCT and ASCT. We report a case of a young man with early intrapulmonary relapse of HD in whom during salvage chemotherapy on interim PET/CT generalized PET-positive lymphadenopathy was observed, caused by a primary Epstein–Barr virus (EBV) infection and not by progressive disease.

## Case presentation

**Figure 1:**
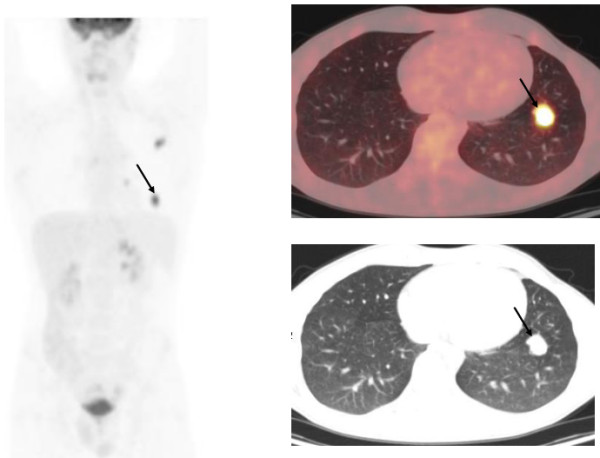
**Interim F**^**18**^**-fluorodeoxyglucose**-**positron emission tomography/computed tomography revealed biopsy/histology-proven intrapulmonary relapse of Hodgkin’s disease after six cycles of Adriamycin® (doxorubicin), bleomycin, vinblastine, dacarbazine and infranodal radiotherapy of a mediastinal mass.** The left image is the total body maximum intensity projection image. The coinciding transverse positron emission tomography and computed tomography slices show the caudal lesion of a total of three lesions in the left lung (black arrows).

A 20-year-old Caucasian man was diagnosed with nodular sclerosing HD stage III-SA with an International Prognostic Score (IPS) of 2 with cervical, supraclavicular, axillary retroperitoneal lymph node localizations, with a bulky mediastinal mass and a splenic nodule on PET/CT with F^18^-fluorodeoxyglucose (FDG). A response evaluation 8 months later after six cycles of Adriamycin® (doxorubicin), bleomycin, vinblastine, and dacarbazine with F^18^-FDG-PET/CT showed a major, both morphologic and metabolic, partial response with minimal focal metabolic activity in his left hilar region for which additional infranodal radiotherapy (in total 15×2Gy) on the mediastinal mass was given. Four months later, after radiotherapy the response evaluation by PET/CT revealed three metabolic active extranodal lesions in his left lung. Relapse of HD was histologically proven using CT-guided biopsy (Figure 1).

Salvage chemotherapy consisted of two cycles of cisplatinum, cytarabine, and dexamethasone (DHAP) and one cycle of etoposide, ifosfamide, and methotrexate (VIM), followed by ASCT with BEAM conditioning was proposed.

Response evaluation using interim F^18^-FDG-PET/CT, after one cycle of DHAP and VIM chemotherapy showed a partial metabolic response in his lung nodes, his maximum standardized uptake value (SUVmax) decreased from 12 and 15 in left upper and lower lobe respectively, to SUVmax 6 and 8.

**Figure 2:**
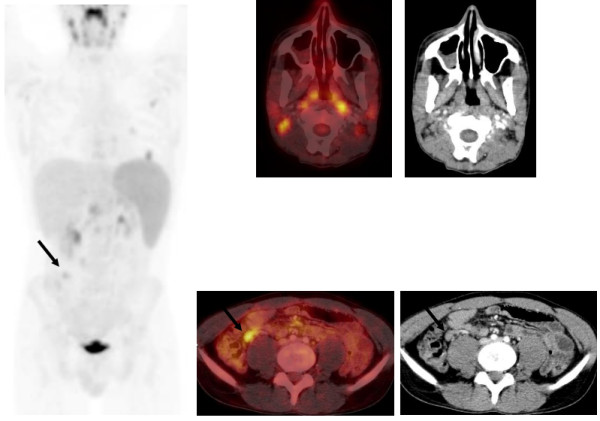
**The second interim F**^**18**^**-fluorodeoxyglucose**-**positron emission tomography/computed tomography after cycles of cisplatinum, cytarabine, and dexamethasone, and etoposide, ifosfamide and methotrexate, showing decreased metabolic activity in the three lesions in the left lung indicating partial response.** However, new pathological increased fluorodeoxyglucose uptake is seen in multiple bilateral cervical plus axillary lymph nodes and furthermore increased metabolic activity in the spleen and in a lymph node within the high-right para-aortal and in a lower-right mesenteric lymph node (black arrows). Virus serology was positive for Epstein–Barr virus.

New symmetrical intense FDG uptake in his adenoids (SUVmax 24), and in multiple bilateral cervical (SUVmax 10), axillary lymph nodes and in a lymph node within the high-right para-aorta (SUVmax 6.5) and a lower-right mesenteric lymph node, with furthermore diffuse increased splenic F^18^-FDG uptake (SUVmax 6) suggested rapidly progressive disease (Figure 2). However, the coincidence of both an extensive nodular relapse with a partial response of the extranodal lung lesions was regarded unlikely. Additional information obtained from the patient revealed he had recently met his first girlfriend. Additional virus serology tests showed EBV-specific immunoglobulin M antibodies and EBV deoxyribonucleic acid (DNA) copies in peripheral blood.

**Figure 3:**
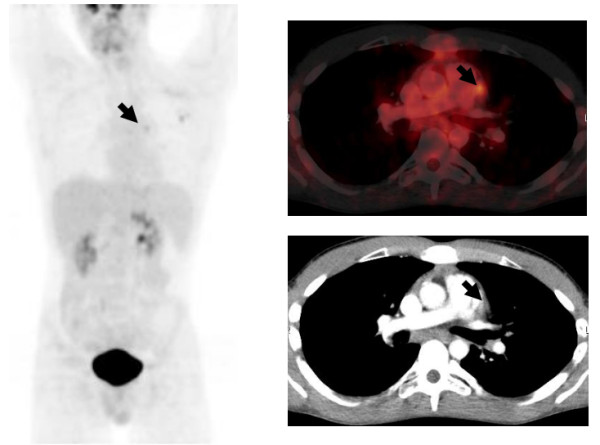
**F**^**18**^**-fluorodeoxyglucose**-**positron emission tomography/computed tomography after completion of chemotherapy with cisplatinum, cytarabine, and dexamethasone-etoposide, ifosfamide, and methotrexate cisplatinum, cytarabine, and dexamethasone and absence of Epstein–Barr virus deoxyribonucleic acid in peripheral blood.** Only the most apical of the three lesions in the left lung is still metabolically active. Minor activity is seen in a small pre-aortal lymph node (thick arrows).

We hypothesized that the asymptomatic primary EBV infection was responsible for the extensive PET-positive lymphadenopathy with a concurrent partial response of HD after DHAP-VIM chemotherapy. After disappearance of EBV DNA copies the second DHAP chemotherapy cycle was given without flare-up of the infection.An interim PET/CT, 2 months after completion of the last DHAP chemotherapy and with absence of EBV DNA in peripheral blood showed metabolic activity in only one of the three lung lesions and only marginal increased activity in a (probably reactive) small mediastinal pre-aortal lymph node (Figure 3). After extensive discussion, he progressed to ASCT and subsequent BEAM conditioning chemotherapy. In the event of extrapulmonary disease progression, he will be considered for treatment with brentuximab vedotin (an antibody-drug conjugate directed to the protein CD30).

## Discussion

EBV is a widely distributed gammaherpesvirus; approximately 90% of adults throughout the world are EBV-positive. Exposure to EBV predominantly occurs in early adulthood. EBV may cause a number of clinical syndromes, including acute mononucleosis and has been associated with a variety of malignancies, including B-cell and T-cell lymphoma [2]. Acute primary infection with EBV is mostly characterized by transient fever, lymphadenopathy and may cause a transient hepatosplenomegaly. The severity and presentation may range from an asymptomatic infection to a serious life-threatening version of mononucleosis with associated liver damage or even rupture of the spleen [3]. Our patient elegantly illustrates that a subclinical primary EBV infection may intervene with both the treatment of HD and remission status judgment. Since PET/CT is rarely used as a diagnostic tool to detect a primary EBV infection in daily clinical practice, only a few case reports have been published. These cases demonstrated that F^18^-FDG uptake can be intense in lymph nodes and spleen and may mimic lymphoma [4-6]. The choice to opt for interim F^18^-FDG PET/CT scans was not only based on the combination of the young age of our patient and the prognostic implications of his relapse. Interim PET is the strongest prognostic factor that is reported for advanced HD, and overshadows the impact of the IPS [7,8]. This case report also supports that better risk stratification, based on early interim (PET) evaluation, may better identify those patients needing intensified chemotherapy. Furthermore, in larger studies the intensification of chemotherapy and the optimal use of PET at the end of chemotherapy have already minimized the use of radiotherapy in advanced disease, thus reducing the risk of long-term complications [9].

## Conclusions

We describe a case with pulmonary relapse of HD, in whom an asymptomatic primary EBV infection was diagnosed during salvage chemotherapy on suggestion of the interim PET/CT. Clinical awareness of intercurrent asymptomatic viral infections, potentially interfering with treatment and remission status monitoring remain important in the interpretation of equivocal medical imaging results.

## Consent

Written informed consent was obtained from the patient for publication of this case report and any accompanying images. A copy of the consent form is available for review by the Editor-in-Chief of this journal.

## Abbreviations

ASCT: Autologous stem-cell transplantation; BEAM: Carmustine, etoposide, cytarabine and melphalan; CT: Computed tomography; DHAP: Cisplatinum, cytarabine and dexamethasone; EBV: Epstein–Barr virus; FDG: F^18^-fluorodeoxyglucose; HD: Hodgkin’s disease; HDCT: High-dose chemotherapy; IPS: International Prognostic Score; PET: Positron emission tomography; SUVmax: Maximum standardized uptake value; VIM: Etoposide, ifosfamide and methotrexate.

## Competing interests

The authors declare that they have no competing interests.

## Authors’ contributions

Data from our patient were collected by HB and MH. MH participated in the clinical management of our patient. The manuscript was prepared by HB and MH. Both authors approved the final manuscript.
